# La verge enfouie congénitale chez l’enfant: à propos d’un cas

**DOI:** 10.11604/pamj.2017.28.296.13864

**Published:** 2017-12-06

**Authors:** Mohamed Rami, Achraf El Bakkaly, Jaouad Bouljrouf, Toualouth Lafia, Mohammed Amine Bouhafs, Rachid Belkacem

**Affiliations:** 1 Service de Chirurgie Urologique Pédiatrique C, Hôpital d’Enfants, CHU Avicenne, Faculté de Médecine, Université Mohammed V, Rabat, Maroc; 2 Service des Urgences Chirurgicales Pédiatriques, Hôpital d’Enfants, CHU Avicenne, Faculté de Médecine, Université Mohammed V, Rabat, Maroc

**Keywords:** Verge enfouie, enfant, Maroc, Buried penis, child, Morocco

## Abstract

La verge enfouie congénitale de l’enfant est une malformation congénitale dans laquelle le pénis semble être de petite taille, mais tous les constituants de la verge sont de taille normale (l’urètre, les corps érectiles et le gland). L’objectif de notre étude était de rapporter notre expérience dans le traitement chirurgical de cette anomalie. Il s’agit d’un nourrisson de 18 mois adressé initialement des urgences à notre service pour prise en charge d’une hydrocèle bilatérale. Cependant, l’examen clinique retrouve une verge enfouie avec un prépuce serré et une dilatation du réservoir préputial par les urines. L’intervention comprend plusieurs étapes: incision en Z, décalottage, libération de la verge par rapport aux adhérences entourant les corps caverneux et couverture cutanée ventrale sur sonde vésicale gardé une semaine pour protéger la cicatrisation. Les résultats esthétique et fonctionnel ont été satisfaisants chez notre patient après un an de recul. La verge enfouie congénitale reste un sujet très débattu. Notre technique était simple et facilement reproductible. Les difficultés mictionnelles, l’infection urinaire sont les indications principales de cette chirurgie.

## Abstract (en)

Congenital buried penis in the child is a congenital malformation where the penis appears small in size while all the parts of the organ are normal (the urethra, the erectile tissue and the glans penis). Our study aimed to describe our experience in the surgical treatment of this abnormality. We report the case of a 18-months old infant with bilateral hydrocele initially admitted to the Emergency Department and then referred to our Department. Clinical examination showed buried penis with tight foreskin and a dilation of the preputial reservoir due to urine. Surgical procedure included several steps: Z-shaped incision, pulling back of the foreskin of the penis, release of the adhesions surrounding the corpus cavernosum and ventral penile skin coverage using bladder catheter kept for a week to protect the wound healing. Aesthetic and functional result was satisfactory after 1-year follow-up. Congenital buried penis is a very debated subject in the literature. Our technique was simple and easily reproducible. Voiding difficulties and urinary infection are the main indications of this surgical procedure.

## Introduction

La verge enfouie congénitale de l’enfant est une pathologie relativement rare et mal connue [1]. Elle est caractérisée par un déficit congénital de longueur de la peau pénienne. Le prépuce peut être serré. Elle est souvent mal diagnostiquée conduisant à une mauvaise circoncision. Le diagnostic est fait devant une taille qui semble réduite de la verge, une miction dans une « chambre préputiale » et des infections urinaires (balanites) [1-3]. L’aspect de verge enfouie se rencontre souvent chez des enfants en surcharge pondérale. Il est dû à la disposition du tissu cutané [4].

## Patient et observation

**Figure 1:**
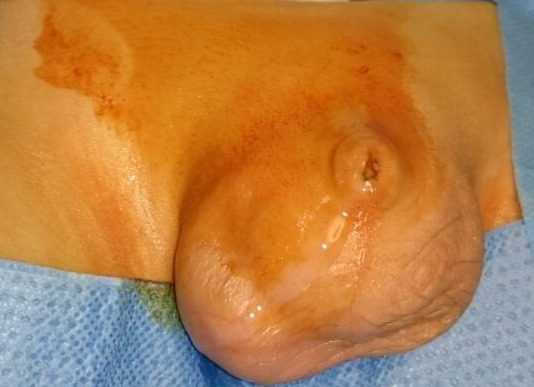
Aspect pré-opératoire de la verge enfouie (mictions sous-préputiales: aspect du prépuce « vide » et « plein »)

Nous rapportons le cas d’un nourrisson âgé d’un an qui a été adressé pour cure d’une hydrocèle bilatérale avec circoncision. Le patient a consulté avec une échographie faite dans un autre établissement, qui parle d’une hydrocèle bilatérale. La mère rapporte également la notion de grosse bourse, mais aussi d’une dysurie faite de miction gouttes à gouttes, deux épisodes d’infections urinaires, et une augmentation variable dans la journée du volume des bourses. A l’examen, nous trouvons un nourrisson en bon état général, apyrétique. L’examen des organes génitaux trouve un enfant non circoncis, avec une tuméfaction prenant la verge et se prolongeant vers les bourses (Figure 1). Nous arrivons à palper la verge sous les tissus. La pression sur cette tuméfaction fait jaillir les urines à travers l’orifice préputial. Le reste de l’examen est normal. Le diagnostic de verge enfouie retenu, le patient fut opéré.

**Figure 2:**
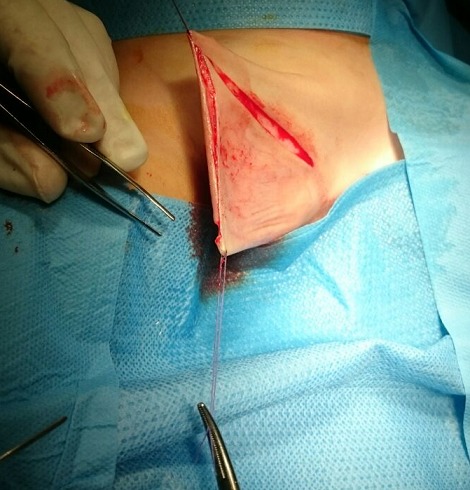
Incision cutanée en Z

**Figure 3:**
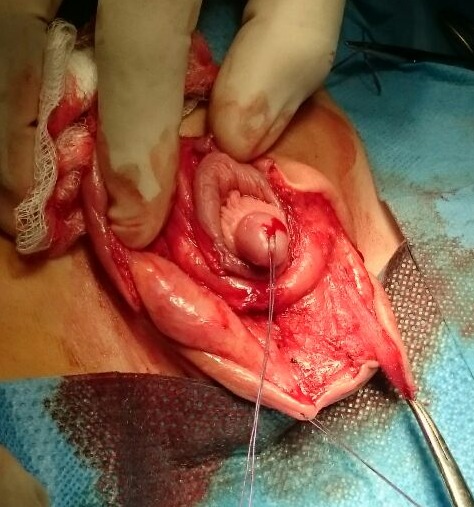
Déshabillage complet de la verge

**Figure 4:**
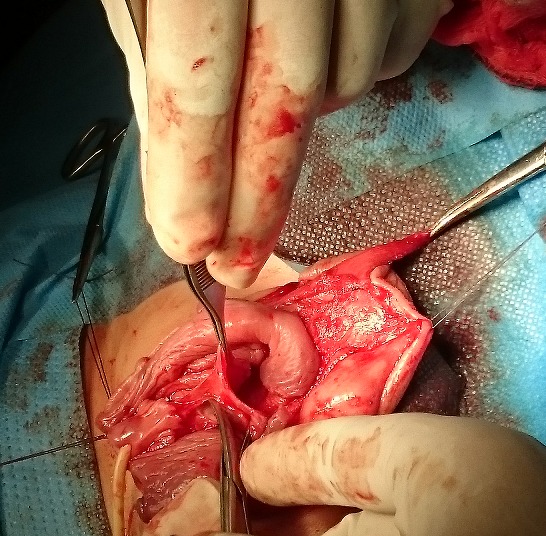
Libération des adhérences entourant les corps caverneux

**Figure 5:**
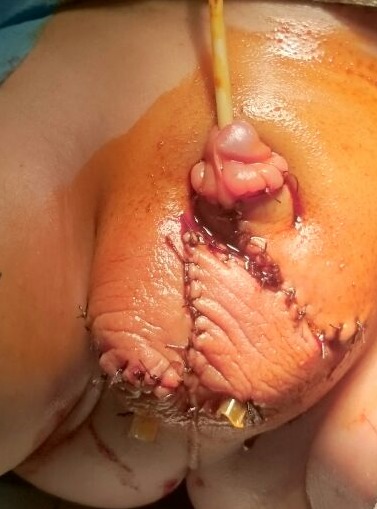
Aspect opératoire final de la verge

**Figure 6:**
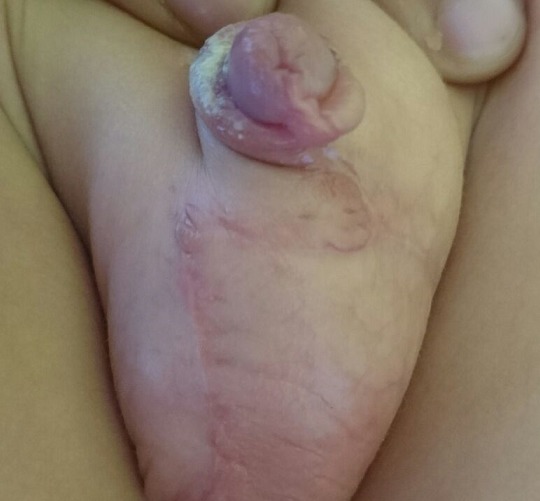
Résultat post-opératoire après un an avec aspect satisfaisant de la verge

L’intervention a consisté en une incision en Z sur la face ventrale de la verge (**Figure 2**), puis un déshabillage total de la verge (Figure 3), la libérant ainsi de toutes attaches et tissus environnants (Figure 4). Un énorme réservoir préputial a été découvert, nécessitant sa résection en laissant une collerette d’environ cinq millimètres. Nous avons procédé ensuite à un amarrage de la base de la verge au fascia de Buck par deux points, puis fermeture de la peau sur la verge (Figure 5). Une sonde urétrale à été laissée pendant cinq jours pour faciliter les soins. Les suites ont été sans complications, le patient fût revu régulièrement en consultation. Après plus d’un an actuellement de la cure chirurgicale, la verge apparaît de taille normale par rapport à l’âge du patient, après réclinaison de la graisse pubienne, la famille est satisfaite du résultat (Figure 6). Le patient sera encore suivi concernant la taille « apparente » de sa verge, on décidera ultérieurement si une autre cure sera nécessaire après diminution de la graisse pubienne.

## Discussion

La verge enfouie est une malformation congénitale, associant un déficit du fourreau cutané de la verge, un défaut de fixation cutanée du fourreau, une obésité avec panicule adipeux prépubien, des adhérences anormales entre le dartos et le fascia de Buck, une fixation inadéquate de la base de la verge ou encore un déplacement anormal du pénis en position ventrale ; alors que la taille de la verge est normale [1,3,4]. La classification de Maizels distingue trois types anatomiques: les verges enfouies, les verges palmées et les verges rétractées, correspondant aux verges enfouies acquises après circoncision [5]. D’autres classifications sont utilisées (Casale et al, Chin et al…) mais aucune n’est universelle [6, 7].

Cliniquement, la famille consulte pour une verge de petite taille, souvent pour des troubles mictionnels: miction discontinue ou goutte à goutte, infection urinaire, apparition d’une tuméfaction qui peut être vidée par pression [6, 8, 9]. La technique chirurgicale préconisée pour notre patient a l’avantage d’être simple et facilement reproductible. En général, et conformément à la technique utilisée dans notre étude, les procédés utilisés par la majorité des chirurgiens pour corriger l´anomalie de la verge enfouie congénitale comprennent le déshabillage complet de la verge, une approche plus directe de la base des corps caverneux en laissant la peau pénienne intacte, la libération des corps caverneux adhérents aux tissus environnants et une couverture cutanée après exérèse de l’excès cutané après ascension des deux lambeaux [10, 11].

Le type d’incision est l’un des points de divergence entre chirurgiens, nous avons choisi l’incision en Z, elle a l’avantage de permettre une bonne exploration et faciliter le déshabillage de la verge ainsi qu’être rapidement fermée mais elle a l’inconvénient comme l’incision dorsale de laisser des cicatrices péniennes parfois inesthétiques. D’autres préconisent l’incision circonférentielle, mais le risque est celui d’une stase lymphatique [11]. Lardellier-Reynaud a choisi dans son étude portée sur 25 enfants, l’incision à la face ventrale de la verge, sans doute parce que la cicatrice est masquée, mais il existait un taux élevé de récidives et des complications [3]. Une autre technique avec incision du raphé scrotal associée à une incision circonférentielle juste en amont du sillon balano-préputial [12] permet l’extériorisation de la verge après déshabillage complet avec une approche plus directe de la base des corps caverneux en laissant la peau pénienne intacte. Au total, En fonction du type d’incision, les résultats sont plus ou moins bons dans la littérature.

La réalisation d’une circoncision simple doit être absolument évitée ou au moins retardée, car elle a un effet esthétique catastrophique. De plus, la circoncision privera le chirurgien de la possibilité d’utiliser la peau préputiale à la fin de l’opération de désenlisement. Celle-ci consiste en un déshabillage complet de la verge, une fixation des fascias aux plans profonds et à une couverture cutanée de novo nécessitant l’utilisation de la peau préputiale. Une lipoaspiration de la graisse sous-cutanée pré-pubienne peut être réalisée [13]. La puberté peut, dans de rares cas, résoudre le problème esthétique. Concernant la prise en charge globale de ces patients, les mesures hygiéno-diététiques destinées à traiter l’obésité constituent un aspect important pour la santé future de ces enfants. En l’absence d’obésité ou en cas de symptomatologie urinaire, la réalisation d’un désenlisement apporte un bénéfice notable au patient. Il n’existe pas de consensus sur le meilleur âge possible [14].

## Conclusion

La verge enfouie congénitale reste un sujet très débattu et nous avons proposé une technique simple et facilement reproductible, avec 96 % de bons résultats sur le désenfouissement [**4**, **7**, **14**]. Enfin, nous rappelons que cette anomalie constitue une contre-indication formelle à la circoncision car elle supprime la peau et la muqueuse nécessaire à la reconstruction [**3**].

## Conflits d’intérêts

Les auteurs ne déclarent aucun conflit d'intérêts.
